# Primary Cutaneous CD30+ Anaplastic Large T Cell Lymphoma in a Patient Treated with Cyclosporine for Actinic Reticuloid

**DOI:** 10.1155/2020/9435242

**Published:** 2020-03-25

**Authors:** T. Gambichler, N. Patsinakidis, L. Susok, M. H. Segert, M. Doerler

**Affiliations:** ^1^Skin Cancer Center, Department of Dermatology, Ruhr-University Bochum, Bochum, Germany; ^2^Division of Experimental Allergy and Immunodermatology, University of Oldenburg, Oldenburg, Germany

## Abstract

Actinic reticuloid (AR)—a subtype of chronic actinic dermatitis—clinically and histopathologically shows lymphoma-like features. We report a male patient initially diagnosed with erythrodermic cutaneous T cell lymphoma (CTCL) who developed severe broadband photosensitivity. Clinical evaluation, histopathology, and phototesting were consistent with AR. The patient was treated with cyclosporine 150–300 mg/d. Under this therapy, he developed several times primary cutaneous anaplastic large cell lymphomas (C-ALCL) which in part tended to regress spontaneously under cyclosporine reduction. The association between cyclosporine treatment and development of C-ALCL and other CD30+ lymphoproliferative disorders has previously been reported in patients with atopic dermatitis, psoriasis, and transplant patients. In conclusion, the present case highlights the difficulties arising in the distinction between AR and CTCL and shows that long-term cyclosporine treatment may cause C-ALCL development in AR as well.

## 1. Introduction

It has been suggested that photosensitive disorders, including persistent light reaction, photosensitive atopic dermatitis, chronic photosensitivity dermatitis, and actinic reticuloid (AR), should be considered as one entity, so called chronic actinic dermatitis (CAD) [1, 2]. This disorder may develop in healthy skin or on the basis of preceding dermatoses, such as atopic dermatitis, photoallergic dermatitis, allergic contact dermatitis, and drug-induced photosensitivity. CAD is defined on three diagnostic criteria as follows: (1) chronic progressive pruritic dermatitis and lichenification predominantly on ultraviolet- (UV-) exposed skin sites, whereby with disease progression, nonexposed sites may also be affected; (2) on UV testing of previously nonexposed skin reduction of the minimal erythema dose (MED) against UV-A and UV-B including the development of eczematous lesions within a few days; and (3) histology consistent with chronic dermatitis, with or without the presence of lymphoma-like changes [1, 2]. Hence, atopic dermatitis, airborne contact dermatitis, and cutaneous T cell lymphoma (CTCL) are the most important differential diagnoses of CAD. UV protection and immunosuppressive therapy including cyclosporine represent standards in the management of CAD or AR [1, 2]. In patients with inflammatory skin conditions, cyclosporine has been reported to be associated with the occurrence of CD30+ lymphoproliferative diseases [3–7]. We here report a difficult-to-diagnose patient with AR under cyclosporine therapy who developed primary cutaneous CD30+ anaplastic large cell lymphoma (C-ALCL) which spontaneously regressed after reduction of cyclosporine dosage.

## 2. Case Presentation

We report a male patient with a history of atopic dermatitis and polyvalent type 1-hypersensitivity since childhood. When he was 46 years old, he had erythroderma, palmoplantar hyperkeratosis, alopecia, and lymphadenopathy. The histologic picture of several skin biopsies showed features of CTCL. Lymph node as well as bone marrow biopsy did not demonstrate evidence for lymphoma manifestation. He was initially treated with high-dose corticosteroids and extracorporeal photopheresis (11 cycles) followed by 9 cycles with liposomal-pegylated doxorubicin. Since he did not adequately respond to doxorubicin, his therapy was switched to bexarotene 150 mg/m^2^ daily.

**Figure 1:**
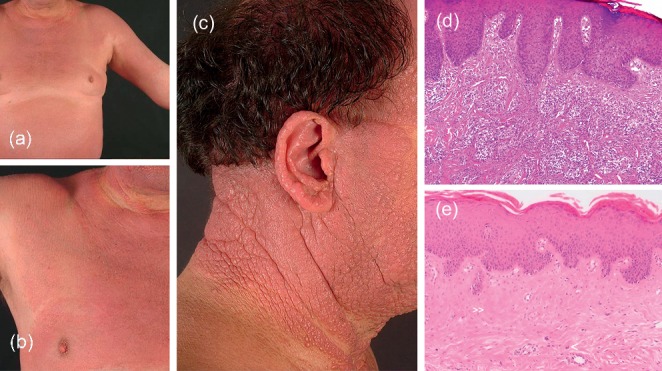
Showing photodistributed erythema (a, b) and massively infiltrated papulous plaques on the face and neck (b) of a patient with actinic reticuloid. Skin biopsy taken from the cheek (c) showing acanthosis and a diffuse lymphocytic infiltrate in the entire dermis. There was no evident epidermotropism of the T cell dominant infiltrate leading to the diagnosis of pseudolymphoma (d). A biopsy taken from eczematous lesions on the trunk (b) showing acanthosis with focal parakeratosis, thickened collagen bundles (>>), and a sparse, mainly lymphocytic infiltrate with hyperchromatic cells (<) in the upper dermis (e). Together, the histologic picture and clinicopathological correlation matched the diagnosis of actinic reticuloid.

At the age of 48 years, he noticed increasing UV sensitivity and worsening of his skin condition, in particular within the sun-exposed skin regions. Notably, he had less skin problems during winter. Clinically, he showed a similar picture as mentioned above. However, the grade of infiltration and induration of skin lesions had significantly increased including impressive tumor-like aggregated papules and nodes mainly affecting the sun-exposed areas (Figure 1). Histology of a papulous lesion on the neck revealed acanthosis and a diffuse lymphocytic infiltrate in the entire dermis. In addition, there was no evident epidermotropism of the T cell dominant infiltrate, resulting in the histologic diagnosis of pseudolymphoma (Figure 1). T cell receptor (TCR) rearrangement was negative. Flow cytometry for lymphocyte subpopulations in the peripheral blood was unremarkable. Again, there was no evidence for lymphoma or other conditions on bone marrow biopsy. Patch-testing including the photopatch test (5 J/cm^2^ UV-A; PUVA 180, Waldmann, Villingen-Schwenningen, Germany) did not reveal evidence for airborne contact dermatitis or photocontact allergy [8]. However, the entire test areal on the lower back showed erythema with eczematous changes following the next days.

**Figure 2:**
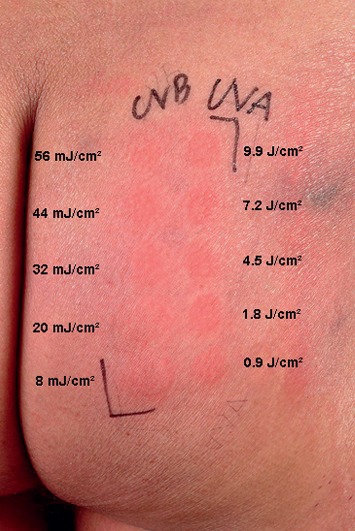
MED testing in a patient with actinic reticuloid showing increased photosensitivity both in the UV-B and UV-A range.

After a brief warm-up period, the MED for broadband UV-B (280–320 nm) and broadband UV-A (320–400 nm) was determined with a compact previously commercially available phototest device (Multitester SBB LT 400; Saalmann GmbH, Herford, Germany). As generally recommended, phototesting was performed on healthy buttock skin [9]. Twenty-four hours after irradiation (Figure 2), the phototest revealed markedly reduced MEDs both for UV-B (<8 mJ/cm^2^) and UV-A (0.9 J/cm^2^) based on previously established lower reference limits of 33 mJ/cm^2^ and 12.6 J/cm^2^, respectively [9]. After 3 days, eczematous lesions were observed in the test sites. Skin biopsies were performed in UV-B as well as UV-A-irradiated sites. Histology revealed dense, in part perivascular or diffuse, infiltrates consisting of monomorph lymphocytes and eosinophils. The infiltrates were predominantly observed in the upper and mid-dermis. On immunohistology, there was a T cell dominant infiltrate (CD8 60%+, CD4 30%+, CD30 negative) and a very low proliferation rate of approximately 5% with Ki-67. TCR rearrangement was negative again. Clinicopathological correlation matched the diagnosis of AR—the lymphoma-like subtype of CAD was made. During the following 9 years, he was predominantly treated with cyclosporine 150–300 mg/d with good control of symptoms, particularly during winter months with almost complete remissions.

**Figure 3:**
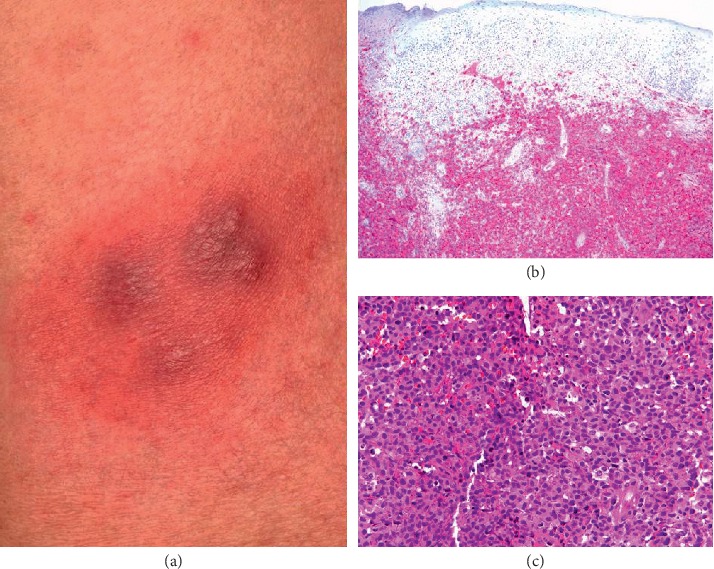
Clinical picture of a histology-proven primary cutaneous anaplastic large T cell lymphoma on the upper right arm (a). CD30 immunohistochemistry showing sheet-like lymphocytic infiltrates of large T cells, which were almost exclusively CD30+ (b, c), consistent with a diagnosis of cutaneous anaplastic large T cell lymphoma.

At the age of 58 years, he re-attended our department because of new reddish ulcerated nodular lesions (3 × 5 cm in diameter) behind the left ear. Histology and immunohistochemistry was consistent with a CD30+ lymphoproliferative disorder. Given the large and polymorphic lymphocytes and the >90% CD30 positivity, a primary C-ALCL was favored. TCR rearrangement was positive. Tests for EBV were negative. Complete work-up including flow cytometry of peripheral blood, bone marrow biopsy, and thoracic/abdominal/pelvic computed tomography showed no evidence for systemic disease. The lesion could only be excised incompletely. Hence, postsurgery radiotherapy was performed. Five months later, he also developed reddish, partly ulcerated nodules on the scalp as well as upper right arm. Again, histology and immunohistochemistry confirmed the diagnosis of C-ALCL (Figure 3). Interestingly, these lesions completely regressed spontaneously about 4 days after the reduction of cyclosporine from 300 mg/d to 100 mg/d. Following further reduction of cyclosporine, it was finally replaced by azathioprine 100 mg daily. No further C-ALCL lesions reoccurred. Importantly, during his 17-year course of disease, there was no evidence for blood abnormalities (e.g., increase of CD3+ CD4+/CD26− T cells) or the presence of monoclonality.

## 3. Discussion

As demonstrated by the present case, the action spectrum of CAD and AR is usually very broad, including UV-B, UV-A, and visible wavelength. Even though not tested in our photodermatology unit, our patient had a history for also being highly sensitive to visible light. The clinical and histopathologic features of AR are similar in nature to those of delayed-type hypersensitivity reaction of the allergic contact dermatitis type. For instance, the observation of increased numbers of cutaneous CD8+ T cells in AR indicates a delayed-type immunologic reaction against a cutaneous antigen (e.g., DNA and RNA) likely induced by UV radiation [1, 2]. To distinguish the severe AR subtype of CAD (previously also called pseudolymphomatous chronic actinic dermatitis) from CTCL, the assessment of TCR rearrangements may be used in addition to immunohistochemistry [10–16]. Particularly in erythrodermic CTCL, the CD4/CD8 ratio is frequently increased as a result of an increase of lesional T helper cells. By contrast in AR, there is frequently an increase of lesional CD8+ cells, frequently resulting in inversion of the CD4/CD8 ratio [13, 15]. In contrast to patients with erythrodermic CTCL, a monoclonal TCR rearrangement is usually not found in the skin or blood of patients with AR. As shown in a previous study on patients (*n* = 231) with CAD, the development of genuine lymphomas was not significantly increased compared with the healthy population [1]. Photosensitivity is, however, an infrequent feature of CTCL as well. Agar et al. [17] suggested that, rarely, malignant clonal T cell populations may recognize a unique UV-induced neoantigen, resulting in the clinical features of severe photosensitivity mimicking those seen in CAD or AR [17]. The long benign course of disease with spontaneous improvement during winter and the absence of significant blood alterations or disease progress widely exclude the diagnosis of CTCL in the present case.

Apart from the fact that AR is a serious mimicker of CTCL, the present case also highlights that patients who are under cyclosporine treatment for inflammatory skin disorders or other conditions are prone to develop C-ALCL. Most patients in such a clinical constellation had atopic dermatitis or psoriasis [18]. Moreover, in organ transplant patients taking immunosuppressants including cyclosporine, the occurrence of CD30+ lymphoproliferative disorders and B cell lymphomas has been observed as well [18–20]. Together, the risk of development of lymphomas in patients with atopic dermatitis or psoriasis on low doses of immunosuppressive therapy (mainly cyclosporine) is considered to be very low [21]. The present case is the first C-ALCL occurrence during cyclosporine therapy for AR. However, our patient previously suffered from long-standing atopic dermatitis that is not infrequently a “precursor” condition of CAD or AR [1, 2]. Indeed, it is barely possible to distinguish C-ALCL from lymphomatoid papulosis (LyP) on histological grounds. Moreover, LyP may manifest clinically with localized or nodular lesions rendering distinction from C-ALCL even more challenging. LyP and C-ALCL, however, differ in their biological behavior and require different treatment approaches. Differentiation between LyP and C-ALCL may succeed by means of immunohistochemistry using markers such as multiple myeloma oncogene 1 and fascin [18–20].

Kirby et al. [7] described a patient with atopic dermatitis who developed C-ALCL during low-dose cyclosporine monotherapy. As similarly observed in the present case, C-ALCL lesions spontaneously resolved after stopping cyclosporine [7]. Fletcher et al. [4] recently reported four cases of CD30+ lymphoproliferative disease in young adult patients with active atopic dermatitis. One of the patients was taking cyclosporine when he developed lymphomatoid papulosis type A that resolved after stopping the drug [4]. Tests for EBV were negative. The authors hypothesize about a link between lymphoproliferative disorder and atopic dermatitis including treatment modalities such as cyclosporine [4]. Possible mechanisms include immunosuppression, chronic antigenic T cell activation, cutaneous bacteria, superantigens, or UV-induced antigens as conceivable in the present case. Cyclosporine inhibits signaling through the TCR to prevent the production of cytokines that would normally stimulate an immune response. This could also theoretically impair T cell activation induced cell death which might prolong lymphocyte survival. Hence, a reduction or cessation of cyclosporine treatment may result in spontaneous regression of CD30+ lymphoproliferative lesions [4, 22].

In conclusion, the present case highlights the difficulties arising in the distinction between AR and CTCL and shows that long-term cyclosporine treatment may cause C-ALCL development in AR as well.
